# Supplementary material for: Systematic characterisation of site-specific proline hydroxylation using hydrophilic interaction chromatography and mass spectrometry
Source: eLife. 2026 Jun 25;14:RP108128. doi: 10.7554/eLife.108128 (PMC13299592; doi:10.7554/eLife.108128)
Supplement: Figure 8—figure supplement 1—source data 1. [file elife-108128-fig8-figsupp1-data1.zip › Figure 8-figure supplement 1-source data 1/Actin-HIF1A-labeled.pdf]

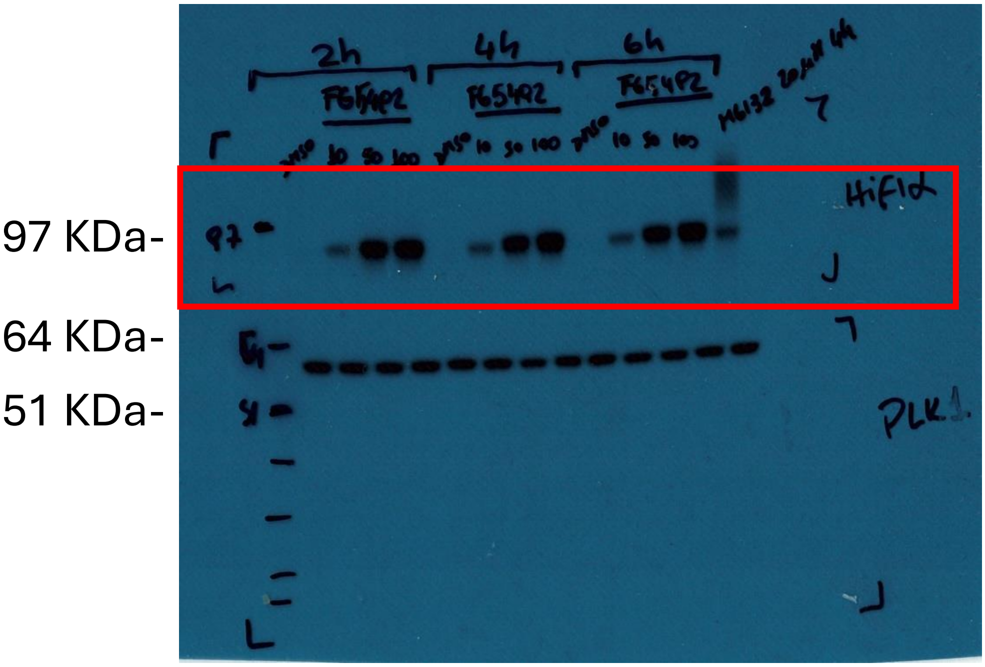

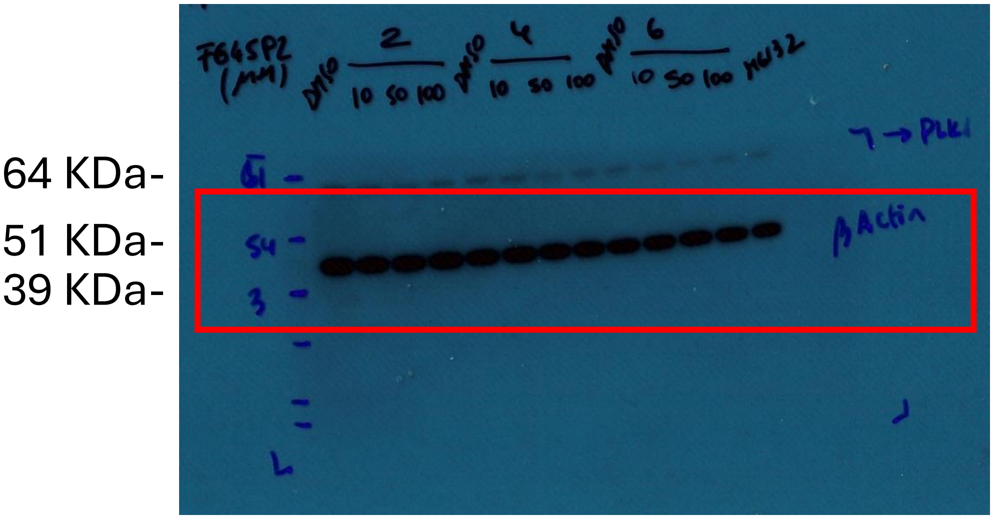

Figure 8-figure supplement 1-source data 1: Original membranes of western blots showing bands including actin and HIF1α (highlighted).
