# Supplementary figures and images for: Systematic characterisation of site-specific proline hydroxylation using hydrophilic interaction chromatography and mass spectrometry
Source: eLife. 2026 Jun 25;14:RP108128. doi: 10.7554/eLife.108128 (PMC13299592; doi:10.7554/eLife.108128)

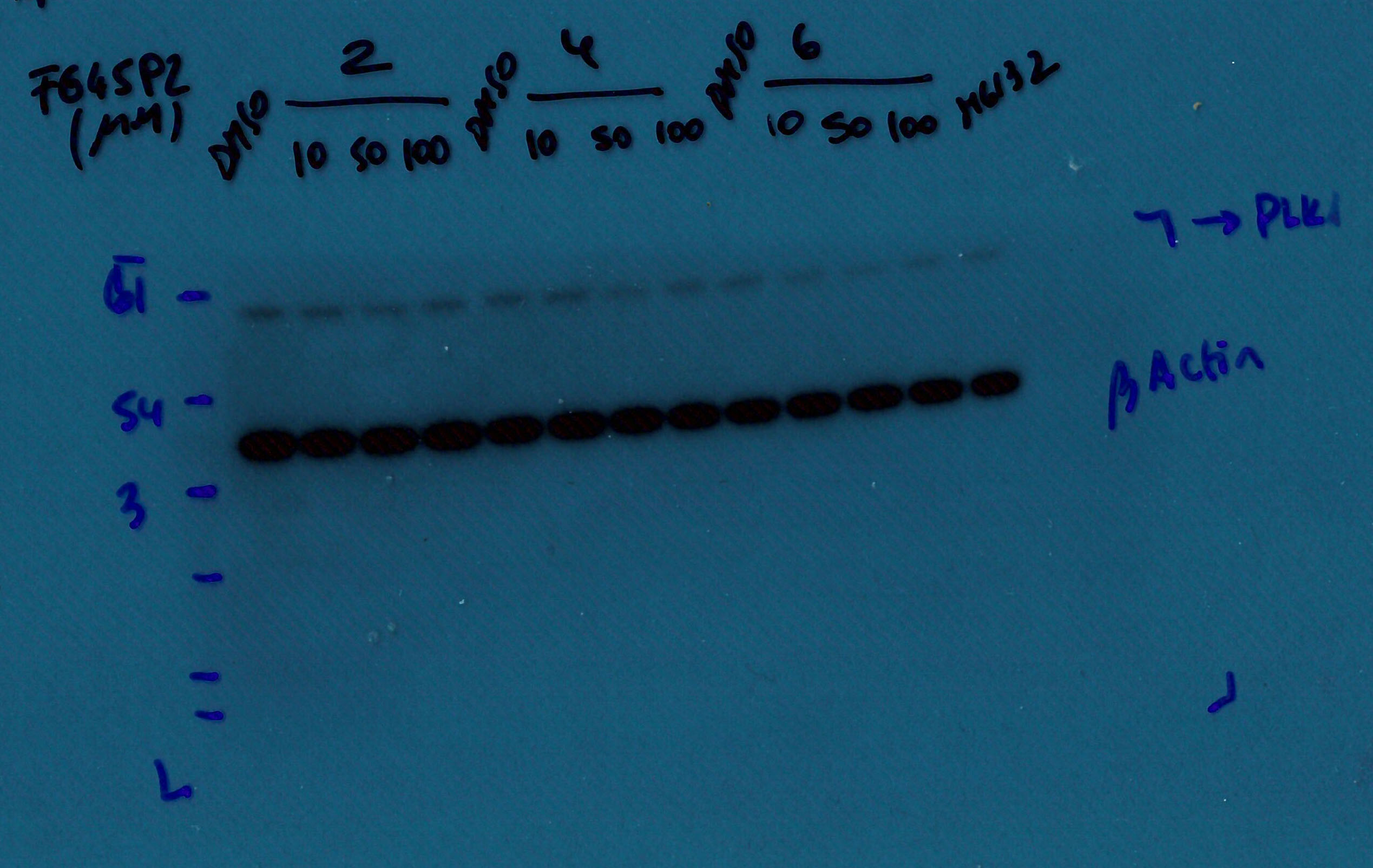

Supplement: Figure 8—figure supplement 1—source data 2. [file elife-108128-fig8-figsupp1-data2.zip › Figure 8-figure supplement 1-source data 2/Actin.jpg]

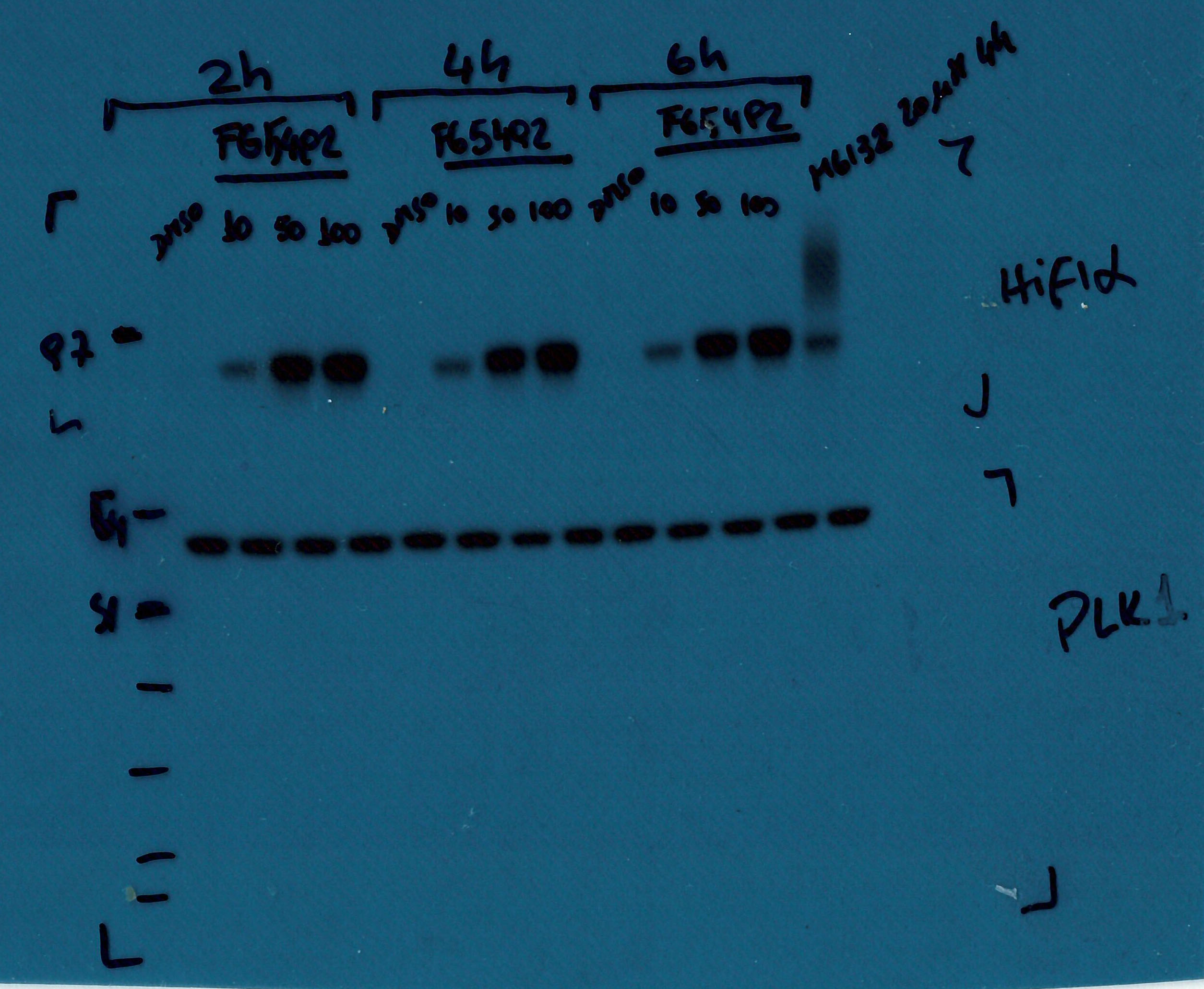

Supplement: Figure 8—figure supplement 1—source data 2. [file elife-108128-fig8-figsupp1-data2.zip › Figure 8-figure supplement 1-source data 2/HIF1A.jpg]

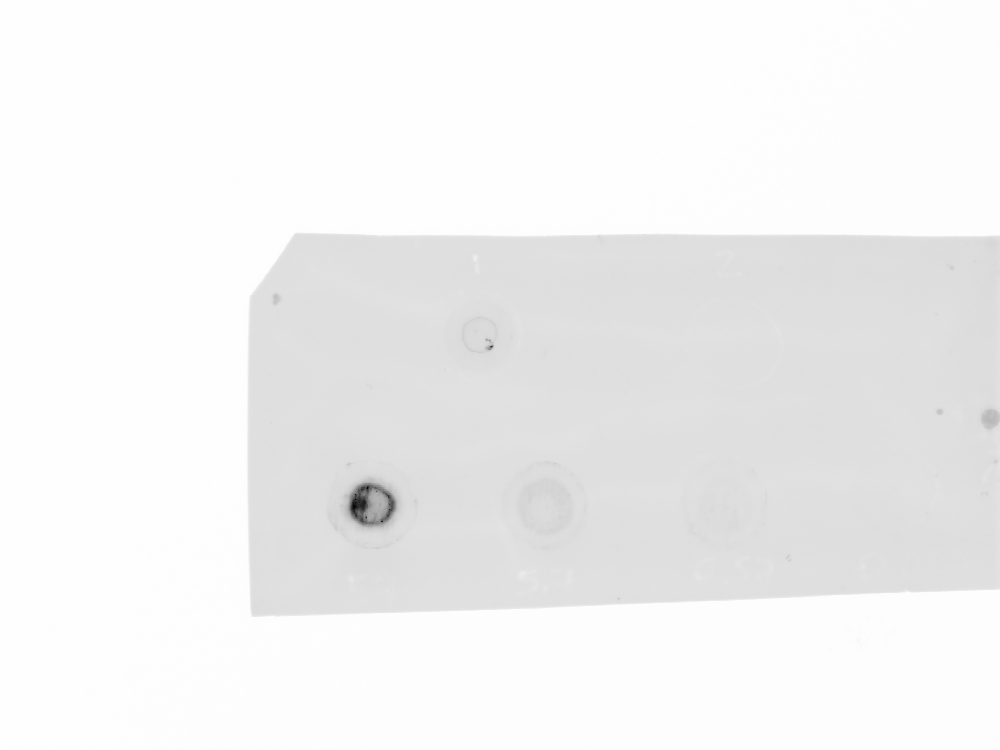

Supplement: Figure 9—source data 2. [file elife-108128-fig9-data2.zip › Figure 9-source data 2/P564OH-HIF1A.tif]
