# Supplementary material for: Systematic characterisation of site-specific proline hydroxylation using hydrophilic interaction chromatography and mass spectrometry
Source: eLife. 2026 Jun 25;14:RP108128. doi: 10.7554/eLife.108128 (PMC13299592; doi:10.7554/eLife.108128)
Supplement: Figure 9—source data 1. [file elife-108128-fig9-data1.zip › Figure 9-source data 1/P564OH-HIF1A-labeled.pdf]

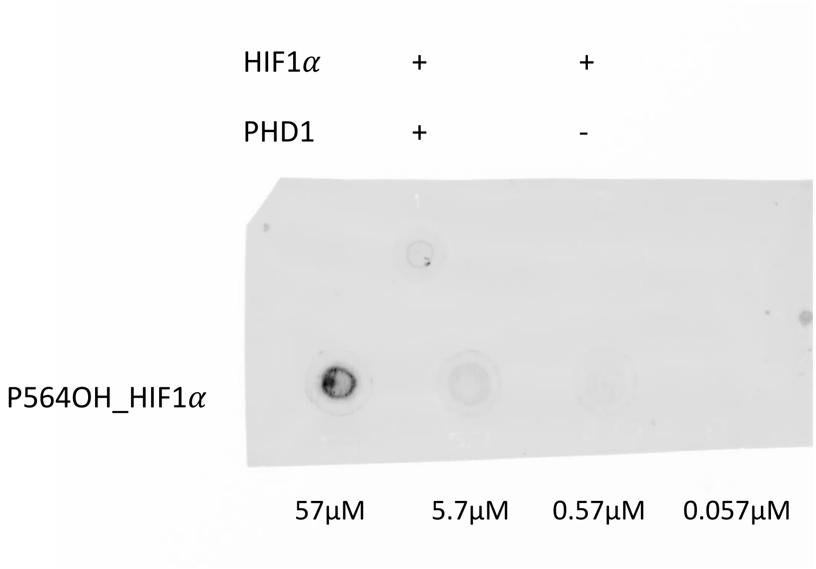

Figure 9-source data 1: Original membranes of dot plots corresponding to Figure 9, panel C, showing conditions with or without PHD1, and different starting amount of synthetic peptides from HIF1α.
