# Supplementary material for: Systematic characterisation of site-specific proline hydroxylation using hydrophilic interaction chromatography and mass spectrometry
Source: eLife. 2026 Jun 25;14:RP108128. doi: 10.7554/eLife.108128 (PMC13299592; doi:10.7554/eLife.108128)
Supplement: Peer review history

# Editor report: eLife Assessment

**DOI:** 10.7554/eLife.108128.3.sa0

**Authors:** Qing Zhang

**Affiliations:** University of Texas Southwestern Medical Center United States

**Keywords:** Solid, Valuable

The study presents a **valuable** resource of proline hydroxylation proteins for molecular biology studies in oxygen-sensing and cell signaling with the characterization of Repo-man proline hydroxylation site. The evidence supporting the claim of the authors is **solid**, although further clarification of the overall efficiency of the HILIC analysis, the specificity/sensitivity of immonium ion analysis, as well as quantification of proline hydroxylation identifications will be helpful. The work will be of interest to researchers studying post-translational modification, oxygen sensing, and cell signaling.

# Referee report: Reviewer #1 (Public review)

**DOI:** 10.7554/eLife.108128.3.sa1

Summary:

The manuscript by Hao Jiang et al described a systematic approach to identify proline hydroxylation proteins. The authors implemented a proteomic strategy with HILIC-chromatographic separation and reported an identification with high confidence of 4993 sites from HEK293 cells (4 replicates) and 3247 sites from RCC4 cells with 1412 sites overlapping between the two cell lines. A small fraction of about 200 sites from each cell line were identified with HyPro immonium ion. The authors investigated the conditions and challenges of using HyPro immonium ions as a diagnostic tool. The study focused the validation analysis of Repo-man (CDCA2) proline hydroxylation comparing MS/MS spectra, retention time and diagnostic ions of purified proteins with corresponding synthetic peptides. Using SILAC analysis and recombinant enzyme assay, the study evaluated Repo-man HyPro604 as a target of PHD1 enzyme.

Strengths:

The study involved extensive LCMS runs for in-depth characterization of proline hydroxylation proteins including four replicated analysis of 293 cells and three replicated analysis of RCC4 cells with 32 HILIC fractions in each analysis. The identification of over 4000 confident proline hydroxylation sites from the two cell lines would be a valuable resource for the community. The characterization of Repo-man proline hydroxylation is a novel finding.

Weaknesses:

As a study mainly focused on methodology, there are some potential technical weaknesses discussed below.

(1) The study applied HILIC-based chromatographic separation with a goal to enrich and separate hydroxyproline containing peptides. The separation effects for peptides from 293 cells and RCC4 cells seems somewhat different (Figure 2A and Figure S1A), which may indicate that the application efficiency of the strategy may be cell line dependent.

(2) The study evaluated the HyPro immonium ion as a diagnostic ion for HyPro identification showcasing multiple influential factors and potential challenges. It is important to note that with only around 5% of the identifications had HyPro immonium ion, it would be very challenging to implement this strategy in a global LCMS analysis to either validate or invalidate HyPro identifications. In comparison, acetyllysine immonium ion was previously reported to be a useful marker for acetyllysine peptides (PMID: 18338905) and the strategy offered a sensitivity of 70% with a specificity of 98%.

(3) The authors aimed to identify potential PHD targets by comparing the HyPro proteins identified with or without PHD inhibitor FG-4592 treatment. The workflow followed a classification strategy, rather than a typical quantitative proteomics approach for comprehensive analysis.

(4) The authors performed inhibitor treatment and in vitro PHD1 enzyme assay to validate that Repo-man can be hydroxylated by PHD1. It remains unknown if PHD1 expression in cells is sufficient to stimulate Repo-man hydroxylation.

# Referee report: Reviewer #2 (Public review)

**DOI:** 10.7554/eLife.108128.3.sa2

Summary:

In this manuscript, Jiang et al. developed a robust workflow for identifying proline hydroxylation sites in proteins. They identified proline hydroxylation sites in HEK293 and RCC4 cells, respectively. The authors found that the more hydrophilic HILIC fractions were enriched in peptides containing hydroxylated proline residues. These peptides showed differences in charge and mass distribution compared to unmodified or oxidized peptides. The intensity of the diagnostic hydroxyproline iminium ion depended on parameters including MS collision energy, parent peptide concentration, and the sequence of amino acids adjacent to the modified proline residue. Additionally, they demonstrate that a combination of retention time in LC and optimized MS parameter settings reliably identifies proline hydroxylation sites in peptides, even when multiple proline residues are present

Strengths:

Overall, the manuscript presents an advanced, standardized protocol for identifying proline hydroxylation. The experiments were well designed, and the developed protocol is straightforward, which may help resolve confusion in the field.

Comments on revisions:

All of my concerns have been resolved by the authors. It is ready for publication.

# Referee report: Reviewer #3 (Public review)

**DOI:** 10.7554/eLife.108128.3.sa3

Summary:

The authors present a new method for detecting and identifying proline hydroxylation sites within the proteome. This tool utilizes traditional LC-MS technology with optimized parameters, combined with HILIC-based separation techniques. The authors show that they pick up known hydroxy-proline sites and also validate a new site discovered through their pipeline.

Strengths:

The manuscript utilizes state-of-the-art mass spectrometric techniques with optimized collision parameters to ensure proper detection of the immonium ions, which is an advance compared to other similar approaches before. The use of synthetic control peptides on the HILIC separation step clearly demonstrates the ability of the method to reliably distinguish hydroxy-proline from oxidized methionine - containing peptides. Using this method, they identify a site on CDCA2, which they go on to validate in vitro and also study its role in regulation of mitotic progression in an associated manuscript.

Weaknesses:

Despite the authors claim about the specificity of this method in picking up the intended peptides, there is a good amount of potential false positives that also happen to get picked (owing to the limitations of MS-based readout), and the authors' criteria for downstream filtering of such peptides requires further clarification. In the same vein, greater and more diverse cell-based validation approach will be helpful to substantiate the claims regarding enrichment of peptides in the described pathway analyses. Experiments must show reproducibility and contain appropriate controls wherever necessary.

Comments on revisions:

I thank the authors for their clarifications and opinions on my questions and suggestions. Based on the response, the following points are important while considering the significance of this manuscript:

- The manuscript provides a novel method to detect and identify proline hydroxylation residues in the proteome. While this provides several advances over previous methods, the probability of false positives, loss of true positives and incomplete removal of the interference of methionine oxidation in this strategy need to be addressed clearly in the discussion section of the manuscript, so that the strengths and limitations of this method are made aware to the reader.

- Going by the standards of publication in eLife, reproducibility is very important in the experiments done. Hence, I strongly recommend that the authors perform the experiments in triplicate with error bars to confirm reproducibility. Graphs with single data points do not convey that, and this is very important for eLife.

- As for Figure 9C, the authors have rejected the request for a control lane in the figure. It may sound trivial to the authors, but for completeness of the experiment, all applicable controls must be performed and shown alongside the main data. It is essential to show the PHD1 only control to rule out the possibility of the contribution of any non-specific signal in the dot blot by PHD1.

# Author comment: Author response

**DOI:** 10.7554/eLife.108128.3.sa4

**Authors:** Hao Jiang, Jimena Druker, James W Wilson, Dalila Bensaddek, Jason R Swedlow, Sonia Rocha, Angus I Lamond

**Affiliations:** University of Dundee Dundee United Kingdom; University of Dundee Dundee United Kingdom; University of Liverpool Liverpool United Kingdom; King Abdullah University of Science and Technology Thuwal Saudi Arabia; University of Dundee Dundee United Kingdom; University of Liverpool Liverpool United Kingdom; University of Dundee Dundee United Kingdom

The following is the authors’ response to the original reviews.

**Public Reviews:**

**Reviewer #1 (Public review):**

Summary:

The manuscript by Hao Jiang et al described a systematic approach to identify proline hydroxylation proteins. The authors implemented a proteomic strategy with HILIC-chromatographic separation and reported an identification of 4993 sites from HEK293 cells (4 replicates) and 3247 sites from RCC4 sites (3 replicates) with 1412 sites overlapping between the two cell lines. From the analysis, the authors identified 225 sites and 184 sites respectively from 293 and RCC4 cells with HyPro diagnostic ion. The identifications were validated by analyzing a few synthetic peptides, with a specific focus on Repo-man (CDCA2) through comparing MS/MS spectra, retention time, and diagnostic ions. With SILAC analysis and recombinant enzyme assay, the study showed that Repo-man HyPro604 is a target of the PHD1 enzyme.

Strengths:

The study involved extensive LC-MS analysis and was carefully implemented. The identification of over 4000 confident proline hydroxylation sites would be a valuable resource for the community. The characterization of Repo-man proline hydroxylation is a novel finding.

Weaknesses:

However, as a study mainly focused on methodology, the findings from the experimental data did not convincingly demonstrate the sensitivity and specificity of the workflow for site-specific identification of proline hydroxylation in global studies.

Proline hydroxylation is an enzymatic post translational protein modification, catalysed by prolyl Hydroxylases (PHDs), which can have profound biological significance, e.g. altering protein half-life and/or the stability of protein-protein interactions. Furthermore, there has been controversy in the field as to the true number of protein targets for PHDs in cells. Thus, there is a clear need for methods to enable the robust identification of genuine PHD targets and to reliably map sites of PHD-catalysed proline hydroxylation in proteins. We believe, therefore, that our methodology, as reported here in Jiang et al., is an important contribution towards this goal. We note that our methodology has already been used successfully by others

(https://doi.org/10.1016/j.mcpro.2025.100969). While further improvements in this methodology may of course be developed in future, we are not currently aware of any superior methods that have been reported previously in the literature. The criticism made by the reviewer notably does not include reference to any such alternative published methodology that interested researchers can use which would offer superior results to the approach we document in this study.

Major concerns:

(1) The study applied HILIC-based chromatographic separation with a goal of enriching and separating hydroxyproline-containing peptides. However, as the authors mentioned, such an approach is not specific to proline hydroxylation. In addition, many other chromatography techniques can achieve deep proteome fractionation such as high pH reverse phase fractionation, strong-cation exchange etc. There was no data in this study to demonstrate that the strategy offered improved coverage of proline hydroxylation proteins, as the identifications of the HyPro sites could be achieved through deep fractionation and a highly sensitive LCMS setup. The data of Figure 2A and S1A were somewhat confusing without a clear explanation of the heat map representations.

The data we present in this study demonstrate clearly that peptides with hydroxylated prolines are enriched in specific HILIC fractions (F10-F18), in comparison with total unfractionated peptides derived from cell extracts. We also refer the reviewer to our previously published study by Bensaddek et al (International Journal of Mass Spectrometry: doi:10.1016/j.ijms.2015.07.029), which was reference 41 in this study, in which we compared directly the performance of both HILIC and strong anionic exchange chromatography, (hSAX). This showed that HILIC provided superior enrichment to hSAX for enrichment of peptides containing hydroxylated proline residues. To clarify this point for readers, we have now included a specific reference to our previous study at the start of the Results section in our current revision. Currently, we use HILIC to provide a degree of enrichment for proline hydroxylated peptides because we are not aware of alternative chromatographic methods that in our hands provide better results.

We have included descriptions of the information shown in the heatmaps in the associated figure legends and captions.

(2) The study reported that the HyPro immonium ion is a diagnostic ion for HyPro identification. However, the data showed that only around 5% of the identifications had such a diagnostic ion. In comparison, acetyl-lysine immonium ion was previously reported to be a useful marker for acetyllysine peptides (PMID: 18338905), and the strategy offered a sensitivity of 70% with a specificity of 98%. In this study, the sensitivity of HyPro immonium ion was quite low. The authors also clearly demonstrated that the presence of immonium ion varied significantly due to MS settings, peptide sequence, and abundance. With further complications from L/I immonium ions, it became very challenging to implement this strategy in a global LC-MS analysis to either validate or invalidate HyPro identifications.

The reviewer appears to have misunderstood the point we make with regard to the identification of the immonium ion and its use as a diagnostic marker for proline hydroxylation in MS analyses. We do not claim that this immonium ion is an essential diagnostic marker for proline hydroxylation. As the reviewer notes, with respect to the acetyl-lysine modification, the corresponding immonium ion is often used in MS studies as a diagnostic for identification of specific post translational modifications. Previous studies have reported that the immonium ion for hydroxylated proline is detected when the transcription factor HIF is analysed, but is often absent with other putative PHD targets, which has been used as an argument that these targets are not genuine proline hydroxylation sites. We are not, therefore, introducing the idea in this study that the hydroxy-proline immonium ion is a required diagnostic marker for proline hydroxylation, but instead demonstrating that detection of this ion, at least in some peptide sequences, may require the use of higher MS collision energies than are typically required for routine peptide identification. We believe that this is an interesting observation that can help to clear up discussions in the literature regarding the true prevalence of PHD-catalysed proline hydroxylation in different target proteins. Our data suggest that, in future MS studies analysing suspected PHD target proteins, two different collision energy might need to be used, i.e., normal collision energy for the routine identification of a peptide, combined with use of a higher collision energy if the hydroxy-proline immonium ion was not already detected.

(3) The study aimed to apply the HILIC-based proteomics workflow to identify HyPro proteins regulated by the PHD enzyme. However, the quantification strategy was not rigorous. The study just considered the HyPro proteins not identified by FG-4592 treatment as potential PHD targeted proteins. There are a few issues. First, such an analysis was not quantitative without reproducibility or statistical analysis. Second, it did not take into consideration that data-dependent LC-MS analysis was not comprehensive and some peptide ions may not be identified due to background interferences. Lastly, FG-4592 treatment for 24 hrs could lead to wide changes in gene expressions and protein abundances. Therefore, it is not informative to draw conclusions based on the data for bioinformatic analysis.

We refer the reviewer to the data we present in this study using SILAC analysis, combined with our MS workflow. to achieve a more accurate quantitative picture of proline hydroxylation levels. While we agree that the point the reviewer makes is valid, regarding our data dependent LC-MS/MS analysis potentially not being comprehensive, this means, however, that we are potentially underestimating the true prevalence of proline hydroxylated peptides, not overestimating the level of these modified peptides. We also refer the reviewer to the accompanying study by Druker et al., (eLife 2025; doi.org/10.7554/eLife.108131.1) in which we present a detailed follow-on study demonstrating the functional significance of the novel proline hydroxylation site we detected in the protein RepoMan (CDCA2). Therefore, even if we have not achieved a fully comprehensive analysis of all proline hydroxylated peptides catalysed by PHD enzymes, we believe that we have advanced the field by documenting a workflow that is able to identify and validate novel PHD targets.

(4) The authors performed an in vitro PHD1 enzyme assay to validate that Repo-man can be hydroxylated by PHD1. However, Figure 9 did not show quantitatively PHD1-induced increase in Repo-man HyPro abundance and it is difficult to assess its reaction efficiency to compare with HIF1a HyPro.

The analysis shown in Figure 9 was not intended to quantify the efficiency of in vitro hydroxylation of RepoMan by PHD1, but rather to answer the question, ‘Can recombinant PHD1 alone hydroxylate P604 on RepoMan in vitro, yes or no?’. The data show that the answer here is ‘yes’. Clearly, the HIF peptide is a more efficient substrate in vitro for recombinant PHD1 than the RepoMan peptide and we have now included a statement in the Discussion that addresses the significance of this observation more directly.

**Reviewer #2 (Public review):**

Summary:

In this manuscript, Jiang et al. developed a robust workflow for identifying proline hydroxylation sites in proteins. They identified proline hydroxylation sites in HEK293 and RCC4 cells, respectively. The authors found that the more hydrophilic HILIC fractions were enriched in peptides containing hydroxylated proline residues. These peptides showed differences in charge and mass distribution compared to unmodified or oxidized peptides. The intensity of the diagnostic hydroxyproline iminium ion depended on parameters including MS collision energy, parent peptide concentration, and the sequence of amino acids adjacent to the modified proline residue. Additionally, they demonstrate that a combination of retention time in LC and optimized MS parameter settings reliably identifies proline hydroxylation sites in peptides, even when multiple proline residues are present.

Strengths:

Overall, the manuscript presents an advanced, standardized protocol for identifying proline hydroxylation. The experiments were well designed, and the developed protocol is straightforward, which may help resolve confusion in the field.

Weaknesses:

(1) The authors should provide a summary of the standard protocol for identifying proline hydroxylation sites in proteins that can easily be followed by others.

This is a good suggestion and we have now included a figure (Figure 10) with a summary of our workflow in the current revision.

(2) Cockman et al. proposed that HIF-α is the only physiologically relevant target for PHDs. Their approach is considered the gold standard for identifying PHD targets. Therefore, the authors should discuss the major progress they made in this manuscript that challenges Cockman's conclusion.

While we had mentioned the Cockman et al., paper in the Introduction, we had not focussed on this somewhat controversial issue. However, in response to the Reviewer’s request, we have now added a comment in the Discussion section in the current revision of how our new data address the proposal discussed previously by Cockman et al. In brief, we believe that our findings are not consistent with a model in which PHDs have no protein targets other than HIFs.

**Reviewer #3 (Public review):**

Summary:

The authors present a new method for detecting and identifying proline hydroxylation sites within the proteome. This tool utilizes traditional LC-MS technology with optimized parameters, combined with HILIC-based separation techniques. The authors show that they pick up known hydroxy-proline sites and also validate a new site discovered through their pipeline.

Strengths:

The manuscript utilizes state-of-the-art mass spectrometric techniques with optimized collision parameters to ensure proper detection of the immonium ions, which is an advance compared to other similar approaches before. The use of synthetic control peptides on the HILIC separation step clearly demonstrates the ability of the method to reliably distinguish hydroxy-proline from oxidized methionine - containing peptides. Using this method, they identify a site on CDCA2, which they go on to validate in vitro and also study its role in regulation of mitotic progression in an associated manuscript.

Weaknesses:

Despite the authors' claim about the specificity of this method in picking up the intended peptides, there is a good amount of potential false positives that also happen to get picked (owing to the limitations of MS-based readout), and the authors' criteria for downstream filtering of such peptides require further clarification. In the same vein, greater and more diverse cell-based validation approach will be helpful to substantiate the claims regarding enrichment of peptides in the described pathway analyses.

We of course agree that false positives may arise, as is true for essentially all PTM studies. There are two issues here; first, are identified sites technically correct? (i.e. not misidentifications from the MS data) and second, are the identified modifications of biological significance? We have addressed this using the popular MaxQuant software suite to evaluate the modifications identified and to control the false discovery rate (FDR) at both the precursor and protein level, as described in the manuscript. We are aware that false positives could arise from confusing oxidation of methionine with hydroxylation of proline. Therefore, to address the issue as to whether we could identify bona fide PHD protein targets outside of the HIF family, we adopted a conservative approach by simply filtering out peptides where there was a methionine residue within three amino acids of the predicted proline hydroxylation site. This was a pragmatic decision made to reduce the likelihood of false positives in our dataset and we recognise that this likely results in us overlooking some genuine proline hydroxylation sites that occur nearby methionine residues. To address the potential biological relevance of the proline hydroxylation sites identified, we analysed extracts from cells treated with FG inhibitors. Of course a detailed understanding of biological significance relies upon follow-on experimental analyses for each site, which we have performed for P604 on RepoMan in accompanying study by Druker et al., (eLife 2025; doi.org/10.7554/eLife.108131.1).

**Recommendations for the authors:**

**Reviewer #1 (Recommendations for the authors):**

(1) The finding that the immonium ion intensities of L/I did not increase with increasing collision energy was surprising. Was this specific to this synthetic peptide?

We agree this is an interesting and unexpected finding. We have no reason to believe that it is specific to synthetic peptides per se, but rather think this reflects an effect of amino acid composition in the peptides analysed. It will be interesting to explore this phenomenon in more detail in future.

(2) The sequence logos in Figure 4 seemed to lack any amino acid enrichment in most positions except for collagen peptides. Have these findings been tested with statistical analysis?

The results we show for sequence logo analysis were generated using WebLogo (10.1101/gr.849004) and correspond to an analysis of all proline hydroxylated peptides we detected across all cell lines and replicates analysed. The fact that collagens are highly abundant proteins with very high levels of proline hydroxylation likely explains why collagen peptides dominated the outcome of the sequence logo analysis. There is clearly scope for more detailed follow up analysis in future of the sequence specificity of proline hydroxylation sites in no- collagen proteins that are validated PHD targets.

(3) Overall figure quality was not ideal. The resolution and font sizes of figures should be carefully evaluated and adjusted. The figure legend should contain a title for the figure. Annotations of the figures were somewhat confusing.

We agree with the criticism of the figure resolution in the review copies - the lower resolution appears to have been generated after we had uploaded higher resolution original images. We are providing again higher resolution versions of all figures for the current revision.

**Reviewer #3 (Recommendations for the authors):**

Certain concerns regarding portions of the manuscript that need addressing:

(1) " These data show that two different cell lines show unique profiles of proteins with hydroxylated peptides." - It is difficult to conclusively say this statement after profiling the prolyl hydroxy proteome from just two cell lines, especially since the amino acids with the highest frequency in the most enriched peptides are similar in both cell lines.

We agree with this point and have changed the current revision to state instead, “This shows that each of the two cell lines analysed have distinct profiles.”

(2) "We noted that there was a high frequency of a methionine residues appearing either at the first, second, or even third positions after the HyPro site.." - according to the authors, claim, the advantage of their method was that they were able to overcome the limitation of older methods that couldn't separate methionine oxidation from proline hydroxylation. However, in this statement, they say that the high frequency of methionine residues may be because of the similar mass shift. These statements are contradictory. The authors should either tone down the claim or prove that those are indeed hydroxyproline sites. Is it possible that in the filtering step of excluding these high-frequency of methionine - containing peptides, we are losing potential positive hits for hydroxy-proline sites? What is the authors' take on this?

We respectfully do not agree that our, “statements are contradictory”, with respect to the potential confusion between identification of methionine oxidation and proline hydroxylation, but acknowledge that we have not explained this issue clearly enough. It is a fact that the similar mass shift resulting from proline hydroxylation and methionine oxidation is a technical challenge that can potentially lead to misidentifications in MS studies and that is what we state clearly in the manuscript. We have addressed this issue head on experimentally in this study and show using synthetic peptides how detailed analysis of specific proline hydroxylation sites in target proteins can be distinguished from methionine oxidation, based upon differential chromatographic behaviour of peptides with either hydroxylated proline or oxidised methionine, as well as by detailed analysis of fragmentation spectra. However, in the case of our global analysis, as we were not able to perform synthetic peptide comparisons for every putative site identified, we took the pragmatic approach of filtering out examples of peptides where a methionine residue was present within three residues of a potential proline hydroxylation site. This was done simply to reduce the possibility of misidentification in the set of novel proline hydroxylated peptides identified and we accept that as a consequence we are likely filtering out peptides that include bona fide proline hydroxylation sites. We have clarified this point in the current revision and hope to be able to address this issue more comprehensively in future studies.

(3) "Accordingly, a score cut-off of 40 for hydroxylated peptides and a localisation probability cut-off of more than 0.5 for hydroxylated peptides was performed." Could the authors shed more light and clarify what was the basis for this value of cut-off to be used in this filtering step? Is this sample dependent? What should be the criteria to determine this value?

We used MaxQuant software (10.1016/j.cell.2006.09.026), for PTM analysis, in which a localization probability score of 0.75 and score cut-off of 40 is a commonly used threshold to define high confidence. The reason that we used 0.5 at the first step was to investigate how likely it might be that the misassignment of delta m/z +16 Da (oxidation) on Methionine would affect the identification of hydroxylation on Proline. However, we note that in the final results set used for analysis, all putative proline hydroxylated peptides that had a Methionine residue near to the hydroxylated proline were disregarded as a pragmatic step to reduce the probability of false identifications.

(4) The authors are requested to kindly make the HPLC and MS traces more legible and use highresolution images, with clearly labeled values on the peaks. Kindly extract coordinates from the underlying data files to plot the curves if needed to make it clearer.

We have reviewed the clarity of all images and figures in the current revision.

(5) There seems to be no error bars in Figure 3, Figure 7E, and panels of Figure 8 with bar graphs. Are those single replicate data?

These specific figures are from single replicate data.

(6) For Figure 9C, the control with only PHD1 (no peptide) is missing.

The ‘no peptide control’ was not included in the figure because it is simply a blank lane and there is nothing to see.
